# Supplementary figures and images for: Rhizosphere Microbiomes of Potato Cultivated under Bacillus subtilis Treatment Influence the Quality of Potato Tubers
Source: Int J Mol Sci. 2021 Nov 8;22(21):12065. doi: 10.3390/ijms222112065 (PMC8584837; doi:10.3390/ijms222112065)

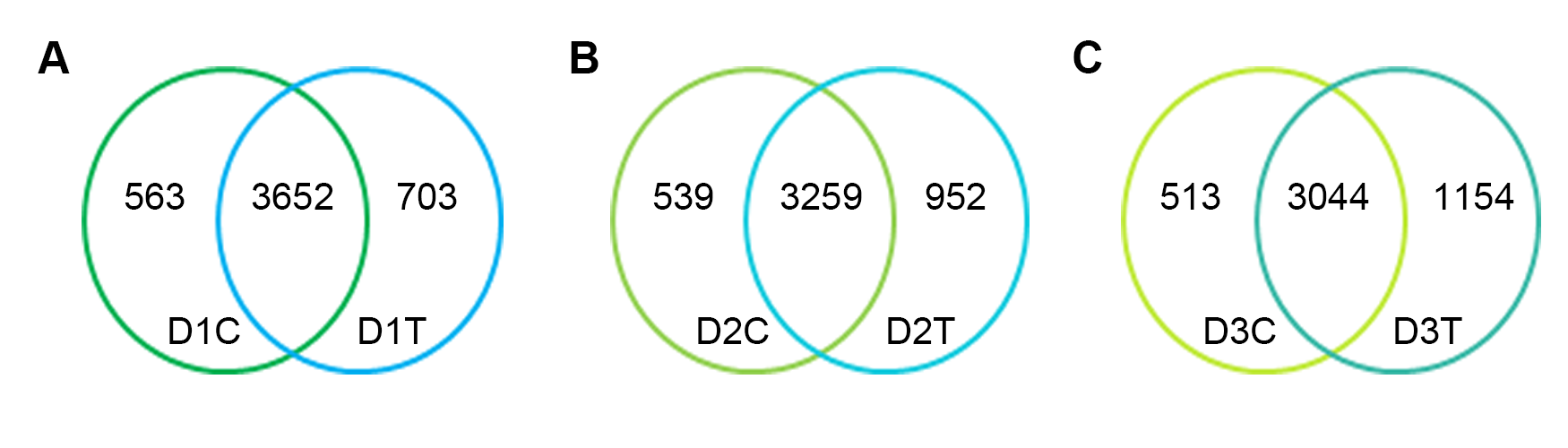

Supplement: Supplementary file 1 [file ijms-22-12065-s001.zip › FigureS1- Venn diagram of bacterial OTUs shows the similarity and differences between treat and control group. (A) Venn diagram of OTUs between D1C and D1T. (B) Venn diagram of OTUs bet.tif]

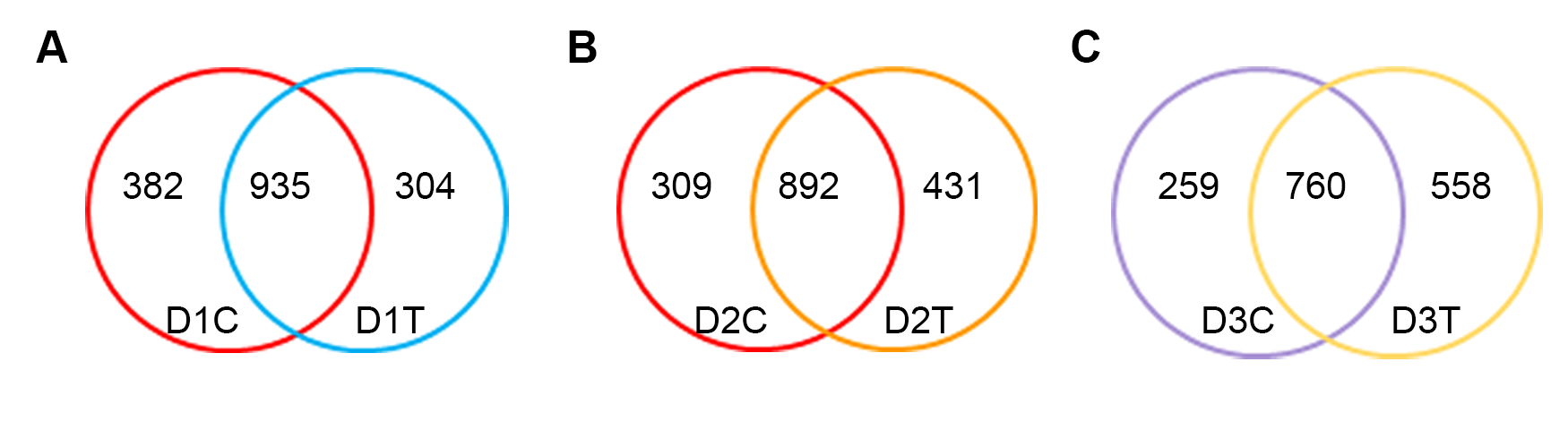

Supplement: Supplementary file 1 [file ijms-22-12065-s001.zip › FigureS2- Venn diagram of fungal OTUs shows the similarity and differences between treat and control group. (A) Venn diagram of OTUs between D1C and D1T. (B) Venn diagram of OTUs betwe.tif]
